# The Individual and Combined Effects of Deoxynivalenol and Aflatoxin B_1_ on Primary Hepatocytes of *Cyprinus Carpio*

**DOI:** 10.3390/ijms11103760

**Published:** 2010-09-29

**Authors:** Cheng-Hua He, Yan-Hong Fan, Ying Wang, Chao-Ying Huang, Xi-Chun Wang, Hai-Bin Zhang

**Affiliations:** 1 College of Veterinary Medicine, Nanjing Agricultural University, Nanjing, 210095, China; E-Mails: hechenghua@njau.edu.cn (C.-H.H.); wangying0624@163.com (Y.W.); huangchaoying@yahoo.cn (C.-Y.H.); 2007207027@njau.edu.cn (X.-C.W.); 2 Wujiang agricultural commission, Wujiang, 215200, China; E-Mail: wittycat@sohu.com (Y.-H.F.)

**Keywords:** deoxynivalenol, aflatoxin B_1_, cytotoxicity, *Cyprinus carpio*, primary hepatocytes

## Abstract

Aflatoxin B_1_ (AFB_1_) and deoxynivalenol (DON) are important food-borne mycotoxins that have been implicated in animal and human health. In this study, individual and combinative effects of AFB_1_ and DON were tested in primary hepatocytes of *Cyprinus carpio*. The results indicated that the combinative effects of AFB_1_ and DON (0.01 μg/mL AFB_1_ and 0.25 μg/mL DON; 0.02 μg/mL AFB_1_ and 0.25 μg/mL DON; 0.02 μg/mL AFB_1_ and 0.5 μg/mL DON) were higher than that of individual mycotoxin (*P* < 0.05). The activity of AST, ALT and LDH in cell supernatant was higher than that of control group (*P* < 0.05) when the mycotoxins were exposed to primary hepatocytes for 4 h. The decreased cell number was observed in tested group by inverted light microscopy. The mitochondrial swelling, endoplasmic reticulum dilation and a lot of lipid droplets were observed in primary hepatocytes by transmission electron microscope. Therefore, this combination was classified as an additive response of the two mycotoxins.

## 1. Introduction

Common carp are native to Europe but have been widely introduced and are now found worldwide except for the poles and northern Asia [1]. Carp are an important food fish throughout most of the world except for in Australia and North America where the fish is considered unpalatable. The world catch rate of carp per year exceeds 200,000 tons.

Aflatoxins are toxic chemicals produced by some species of naturally occurring moulds (*Aspergillus flavus* and *Aspergillus parasiticus*). Aflatoxins are common contaminants of oilseed crops such as cottonseed, peanut meal, and corn. Wheat, sunflower, soybean, fish meal, and nutritionally complete feeds can also be contaminated with aflatoxins, especially in warm and humid climates [2–5]. Four major aflatoxins (AFB_1_, AFB_2_, AFG_1_ and AFG_2_) are direct contaminants of grains and finished feeds. AFB_1_ is one of the most potent, naturally occurring, cancer-causing agents in animals. Aflatoxicosis is a disease that can affect many species of fish when feed contaminated with aflatoxins is eaten by the fish [6]. The first documented incidences of aflatoxicosis affecting fish health occurred in the 1960s in trout hatcheries [7]. The carcinogenic effect of AFB_1_ has been studied in fishes such as salmonid, rainbow trout, channel catfish, tilapia, guppy and Indian *major carps* [8–13].

Deoxynivalenol (DON) is prevalent worldwide in crops used for food and feed production, including in Canada and the United States [14]. Although DON is one of the least acutely toxic trichothecenes, it should be treated as an important food safety issue because it is a very common contaminant of grain. Toxic effects of DON in animals have been well documented and focused mainly on the immune system and the gastrointestinal tract. In particular, the symptoms of acute toxicosis at high dosage included diarrhea, vomiting, leukocytosis, hemorrhage, circulatory shock and ultimately death. Chronic toxicosis would lead to anorexia, reduced weight gain, nutrients malabsorption, neuroendocrine changes and immuno-suppress [15].

AFB_1_ and DON are important food-borne mycotoxins. They occur simultaneously in food including aquatic feed [16]. The use of more plant-based ingredients in aquafeeds enhances both the risk of introducing mycotoxins into the feed at the point of feed manufacturing, and mycotoxin production during storage of compounded feed [17]. After the fish feed were contaminated by fungi, more than one mycotoxin existed. In China, the AFB_1_ and DON were the most common mycotoxins in fish feed. In this research, the individual and combined effects of DON and AFB_1_ in primary hepatocytes of *Cyprinus carpio* were reported.

## 2. Results and Discussion

### 2.1. Individual and Combined Toxicity of DON and AFB_1_ on Primary Hepatocytes

**Figure 1:**
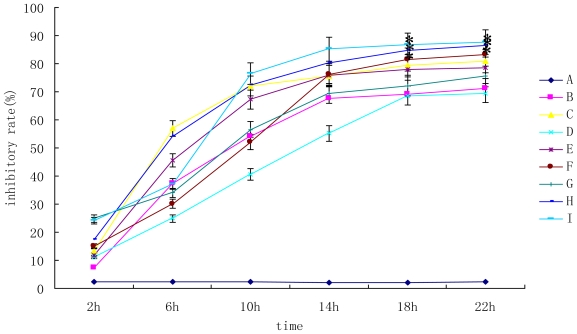
The individual and combined inhibitory rate of DON and AFB_1_ on primary hepatocytes. A, control; B, 0.01 μg/mL AFB_1_; C, 0.02μg/mL AFB_1_; D, 0.25 μg/mL DON; E, 0.5 μg/mL DON; F, 0.01 μg/mL AFB_1_ and 0.25 μg/mL DON; G, 0.01 μg/mL AFB_1_ and 0.5 μg/mL DON; H, 0.02 μg/mL AFB_1_ and 0.25 μg/mL DON; I 0.02 μg/mL AFB_1_ and 0.5 μg/mL DON. * means significant difference at 0.05 level (*P* < 0.05). The same as follows.

To evaluate the influence of the mycotoxins on primary hepatocytes, the MTT assay was used. The results were shown in Figure 1. The results showed that the toxicity of mixture (group F, H and I) was higher than that of individual mycotoxin (*P* < 0.05). Group I was the most toxic (cell inhibitory rate was 87.67%). After the mycotoxins added, the inhibitory rate increased with the time lasted and increasing mycotoxin concentration. However, the concentration-response curves were not clear.

### 2.2. The Enzyme Activity of AST ALT and LDH in Cell Supernatant

**Figure 2:**
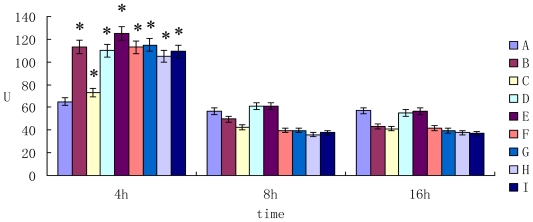
The activity of AST in cell supernatant.

The activity of aspartate aminotransferase (AST) in cell supernatant was shown in Figure 2. When the mycotoxin was exposed to primary hepatocytes for 4 h, the activity of AST was higher than that of control group (*P* < 0.05). However, the activity of AST decreased after 8h and 16h incubation (*P* > 0.05) compared to the control group.

**Figure 3:**
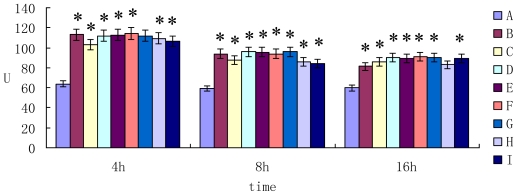
The activity of ALT in cell supernatant.

The activity of alanine transarninase (ALT) in cell supernatant was shown in Figure 3. The results showed that the activity of ALT in mycotoxin group was higher than that of control group after 4 h, 8 h and 16 h incubation (*P* < 0.05).

**Figure 4:**
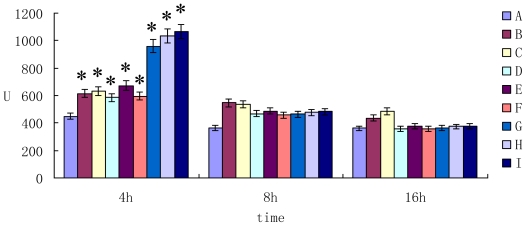
The activity of LDH in cell supernatant.

The activity of lactate dehydrogenase (LDH) in cell supernatant was shown in Figure 4. The results showed that the activity of LDH in mycotoxin group was higher than that of control group after 4 h incubation (*P* < 0.05). However, prolonging the cultured time (for 8 h and 16 h incubation), the difference of the activity of LDH between the groups was not significant (*P* > 0.05).

### 2.3. Morphological Observation on Cells

**Figure 5:**
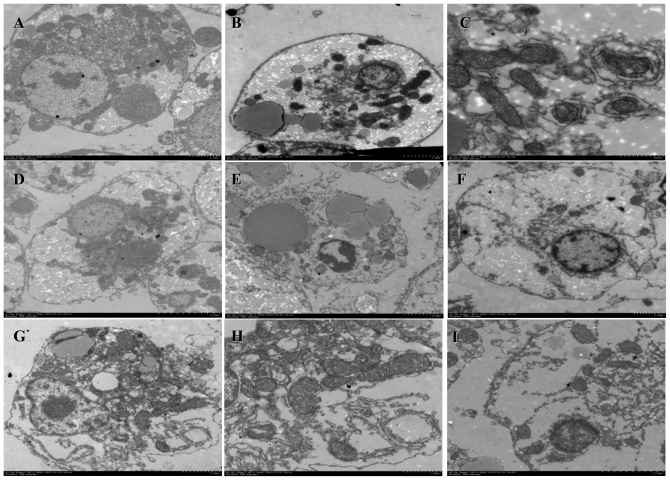
The ultrastructure of primary hepatocytes.

The ultrastructure of primary hepatocytes was shown in Figure 5. The cell membrane of normal hepatocytes was intact. The intranuclear chromatins were homogeneous granules. The organelles were clearness (Figure 5A). When the primary hepatocytes were cultured with the AFB_1_ (0.01 μg/mL), the lipid droplets, nucleus and chromatin condensation were observed clearly (Figure 5B). The saccular ectasia and vesicular structure were observed in rough endoplasmic reticulum and the smooth endoplasmic reticulum, respectively (Figure 5C). There were a lot lipid droplets and shrinkage nucleuses in hepatocytes (Figures 5D, 5E). The mitochondrial swelling was observed in hepatocytes (Figure 5F). The nuclear membrane dissolves and the endoplasmic reticulum dilation were observed in hepatocytes (Figures 5G, 5H, 5I).

AFB_1_ and DON always occur simultaneously in aquatic feed. They play an important role in the mycotoxicoses of *Cyprinus carpio*. In order to analyze the predictability of the combined effects of a mixture of AFB_1_ and DON, it is necessary to examine the individual effect of each mycotoxin on primary hepatocytes of *Cyprinus carpio*. The results showed that the combined toxicity of AFB_1_ and DON (Group F, H and I) was higher than that of individual mycotoxin (*P* < 0.05). The toxic effects included inhibition of cell growth, destruction of cell structure and increasing activity of AST, ALT and LDH. Up to now, many reports were focused on the single mycotoxin on cells [18–23]. The principal target organ for aflatoxins was the liver. After the invasion of AFB_1_, it would lead to necrosis or death of liver cells. In the experiment, we found that AFB_1_ could lead to primary hepatocytes death. There were a few reports about the toxicology of combinative mycotoxin [24–27]. The toxicity of AFB_1_ and DON on the injury and repair of DNA was studied [28]. The report only showed that AFB_1_ and DON produced marked effects on engendering tumor as genetoxic and nongenotoxic carcinogens complementarily.

The AST, ALT and LDH were secreted into cell supernatant from liver cells when they were destroyed. The activity of these enzymes only lasted for 4 h. So, these enzymes should be tested immediately. When primary hepatocytes were cultured with mycotoxins for 4 h, the activity of these enzymes increased significantly. However, the activity of these enzymes decreased at 8 h and 16 h, because almost primary hepatocytes were dead.

Many apoptotic cells were observed in individual mycotoxin group, however, a lot of necrotic cells in combinative group. The all results could indicate that a combined intake of mycotoxins would lead to a possible higher risk for adverse health effects than the intake of one of these mycotoxins alone. However, the data on combined toxic effects of mycotoxins were generally limited, particularly with respect to the AFB_1_ and DON. The further research would focus on the mechanism of apoptosis and necrosis induced by the AFB_1_ and DON.

## 3. Experimental Section

### 3.1. Mycotoxin

AFB_1_ and DON were purchased from Sigma–Aldrich Co. (mainland, China) and dissolved in dimethylsulfoxide (DMSO, Merck, Darmstadt, Germany).

### 3.2. Cell Cultures and Treatments

The method of isolation of primary hepatocytes was performed following the reference[29]. The primary hepatocytes were cultured in L-5 medium at 27 °C in CO_2_ incubator without CO_2_. Cells were seeded at a density of 2 × 10^6^ cells/mL in 24-well tissue culture plates. The experiment was designed with two factors and three levels. The final concentration of AFB_1_ and DON was 0 μg/mL, 0.01 μg/mL, 0.02 μg/mL and 0 μg/mL, 0.25 μg/mL, 0.5 μg/mL, respectively. The DMSO (2 μL/mL, final concentration) was the control group. Each group has 48 duplications.

### 3.3. Cytotoxicity Assay by MTT Test

The cytotoxicity were determined using the colorimetric method described by T.Mosmann [30]. Cells were seeded on 96-well culture plates (Polylabo, France) at 2 × 10^6^ cells/mL and treated with different concentrations of AFB_1_ or DON and/or AFB_1_ + DON combined for 2, 6, 10, 14, 18, 22 h at 27 °C, respectively. Then, the medium was moved carefully from the wells and the wells were washed once with PBS. MTT (concentration 5 mg/mL in PBS; 20 μL/well) was then added to the wells. The cultured cells were returned to the incubator for 4 h. The medium was discarded and the wells were dried. Formazan crystals produced were solubilized using 100 μL dimethyl sulfoxide (DMSO)/well and misce bene. Absorbance was measured in a Stat Fax 2000 ELIASA (Bio-Rad, USA) at 570 nm wavelength, to assess cell proliferation of the primary hepatocytes. The results were expressed as:

Inhibitory rate=(1-mean OD mycotoxin in sample/mean OD solvent control)×100%

### 3.4. The Enzyme Activity of AST, ALT and LDH in Cell Supernatant

After the mycotoxin added, the cell supernatant was collected at 4 h, 8 h and 16 h, respectively. The enzyme activity of AST, ALT and LDH was detected in cell supernatant by automated biochemical analyzer (HITACHI 7150, Japan).

### 3.5. Morphological Observation on Cells

The growth condition was observed by inverted light microscopy (Nikon, Japan). After the primary hepatocytes incubation with mycotoxins for 24 h, the cells were collected and fixed with 2.5% glutaraldehyde (pH 7.4). The ultrastructure of cell was observed by transmission electron microscope (HITACHI H-7650).

### 3.6. Data Analysis

Data were analyzed by SPSS 12.0 with One-Way ANOVA mode.

## 4. Conclusions

The experiments reported here were conducted to evaluate the effect of these toxins on primary hepatocytes. The results indicated that the combinative toxicity of AFB_1_ and DON (Groups F, H and I) was higher than that of individual mycotoxin (*P* < 0.05). However, what we research was the single mycotoxin on the cells. Therefore, we should more focus on the combinative mycotoxins on animal and human health.
